# Understanding Genotype-Phenotype Effects in Cancer via Network Approaches

**DOI:** 10.1371/journal.pcbi.1004747

**Published:** 2016-03-10

**Authors:** Yoo-Ah Kim, Dong-Yeon Cho, Teresa M. Przytycka

**Affiliations:** National Center for Biotechnology Information, National Library of Medicine, National Institutes of Health, Bethesda, Maryland, United States of America; Johns Hopkins University, UNITED STATES

## Abstract

Cancer is now increasingly studied from the perspective of dysregulated pathways, rather than as a disease resulting from mutations of individual genes. A pathway-centric view acknowledges the heterogeneity between genomic profiles from different cancer patients while assuming that the mutated genes are likely to belong to the same pathway and cause similar disease phenotypes. Indeed, network-centric approaches have proven to be helpful for finding genotypic causes of diseases, classifying disease subtypes, and identifying drug targets. In this review, we discuss how networks can be used to help understand patient-to-patient variations and how one can leverage this variability to elucidate interactions between cancer drivers.

Teresa M. Przytycka is an Associate Editor of *PLOS Computational Biology*.

## Introduction

Biological networks provide a natural representation of complex biological systems and thus have been used in a variety of applications, from gene function prediction to identifying disease genes. In particular, complex diseases such as cancer can be better understood from the perspective of dysregulated pathways, rather than as a disease resulting from alterations of individual genes. One of the most popular types of biological networks used in disease studies is a gene interaction network. In a gene interaction network, genes are represented as nodes, and edges connect pairs of genes that are physically interacting or functionally related. Physical interaction networks can be constructed based on physical interactions, such as protein-protein interactions, protein-DNA interactions, and phosphorylation [1–3]. Functional interaction networks connect genes with similar or related functions and are typically inferred from multiple sources, including physical interactions, co-expression, Gene Ontology (GO) terms, etc. [4,5]. Other types of networks that are also considered in disease studies are patient similarity networks [6–8], disease-phenotype networks [9–13], and drug target networks [14].

Interaction networks can be generic or condition-dependent. Generic networks, such as protein-protein interaction networks, summarize existing knowledge of the system that is typically not state or tissue-specific. Such static interaction maps between genes provide a scaffold for disease studies. Combined with additional disease related features, they can guide the identification of genes and pathways affected by a disease. For example, gene expression data have been shown to be useful for identifying network markers of a disease in the form of connected subnetworks that have significantly differential gene expression in two disease phenotypes [15–20]. Importantly, it has been demonstrated that such network markers can lead to a more robust disease classification relative to classification based on individual genes [15]. Subsequently, many different algorithms have been developed to identify gene modules that are either differentially expressed between different disease states or show similar expression patterns. Network-based algorithms have also been used to explain expression Quantitative Trait Loci (eQTL) relationships and infer causal pathways through which genetic alterations affect gene expression changes [21,22].

One of the critical and challenging problems in disease studies is the identification and explanation of genotype-phenotype relationships. In cancer datasets, the relationships between genetic alterations and phenotypes are typically not one-to-one. Different genetic aberrations in different cancer patients can lead to the same disease phenotype, which makes it difficult to uncover genotype-phenotype relationships. One explanation of the heterogeneity between cancer cases is that different genetic alterations can dysregulate the same pathways, resulting in similar disease phenotypes. A network-centric view of diseases helps overcome the challenges posed by the complex genotype-phenotype relationships and facilitates finding genotypic causes of diseases [15,20,21]. It is worth noting that in addition to intertumor heterogeneity, variability between cells within individual tumors has been observed. Such intratumor heterogeneity has typically been studied using different approaches such as phylogeny-based methods.

Another challenge in cancer studies relates to differentiating cancer-driving mutations from passenger mutations. It is generally assumed that while somatic cancer cells typically contain many genomic aberrations, only a few of them are driver mutations, i.e., mutations that provide a growth advantage for tumor cells and thus are causal with respect to cancer progression. In contrast, the mutations that do not confer growth advantages are referred to as passenger mutations. For example, linkage disequilibrium accompanying some types of genomic aberrations, such as copy number variations, results in many passenger mutations. In addition, cancer cell divisions accumulate random mutations—most of them passengers. A faulty gene in the DNA repair mechanism or environmental factors such as UVC exposure can result in both passenger and driver mutations. Identification of drivers among such mutations is fundamental to cancer studies. While a high mutation rate in cancer samples is an indicator of possible driver activity, information on mutation rates alone does not suffice to correctly identify all cancer drivers. It is believed that there are many cancer driving mutations that occur at relatively low frequency, making it difficult to distinguish them from background noise. By placing the mutated genes in the context of other genes and identifying closely connected clusters of mutated genes in interaction networks, network approaches have been used to find defective gene modules in complex diseases such as cancer, autism, and autoimmune diseases [23–27].

Several large-scale, cancer-related datasets, such as the data generated by TCGA (The Cancer Genome Atlas, http://cancergenome.nih.gov) and ICGC (International Cancer Genome Consortium, https://icgc.org), are currently available. These publicly available datasets offer comprehensive genomic profiles, including gene expression, somatic mutations, copy number variations, and DNA methylation, for an unprecedented number of patients. Integration of such datasets together with interaction networks can offer great opportunities to advance our understanding of the cause of diseases, classification of disease subtypes, and identifying drug targets [15–22].

In this review, we outline the basic strategies of network-based approaches in the analysis of cancer datasets, mostly focusing on methods that deal with intertumor heterogeneity. In the first few sections, we discuss how gene interactions can be used to overcome difficulties due to cancer heterogeneity and find cancer driving genes (Network Propagation to Uncover Cancer Driving Pathways and Heterogeneity of Cancer and Personalized Networks). The mutual exclusivity relationships that emerge because of such heterogeneity and the methods utilizing such relationships are reviewed in Taking Advantage of Cancer Heterogeneity: Utilizing Mutual Exclusivity of Genomic Alterations. In the following sections, we discuss other types of disease-related networks, such as patient similarity networks (Patient Similarity Networks) and disease phenotype networks (Disease Similarity Networks).

## Network Propagation to Uncover Cancer Driving Pathways

A single mutation in a gene is often enough to perturb an entire pathway. Furthermore, a relatively small number of pathways are affected in cancers [23,28–30]. Driver genes (i.e., genes harboring driver mutations) are therefore expected to be sparsely located in a gene network when an individual patient is considered, but the driver genes influence genes in its neighborhood. Methods utilizing information propagation are particularly well suited for such settings since they facilitate detecting commonly influenced groups of genes. Variations of the information propagation technique include heat diffusion, network smoothing, random walk, and circuit flow algorithms. These approaches have been used in many applications, such as disease gene prioritization [31,32], gene function prediction [33–35], gene-disease association [36], and finding network centrality [37,38].

Network propagation techniques have been used in several different applications. One application is to uncover causal paths linking perturbed causal genes to other affected genes [39–43]. In general, the approach tests whether a genetic perturbation in a particular locus (or a drug effect on a particular protein) is likely to affect expression of a specific gene or genes of interest. In addition, finding most probable causal paths allows for uncovering which other genes are likely to participate in propagating this information.

**Fig 1:**
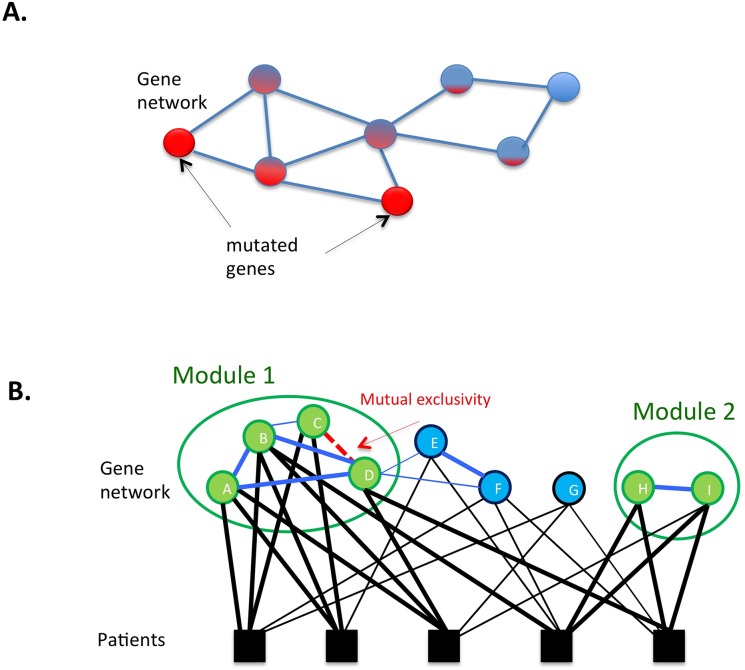
Illustrations of how gene networks can be used in cancer data analysis. A) Information propagation approach: the information about mutated genes (in red) is propagated to their neighborhood through interactions, helping to identify significantly affected subnetworks. The level of redness of a node indicates how likely the gene is affected. B) Module Cover approach finds the minimum cost subnetworks so that each patient is covered by at least k mutated genes. The edges in the gene interaction network (blue edges) may be weighted based on interaction confidence or mutual exclusivity. For example, the patients covered by gene C and D are mutually exclusive. There is an edge between a gene and a patient if the gene is mutated in the patient (black edges). The figure shows an example where two modules are selected, covering each patient at least three times (k = 3). The green nodes are selected genes, and the thick edges indicate the selected interactions or gene-patient relationships.

Another application is to combine information on a large set of patients to identify frequently (or differentially) mutated subnetworks that are likely to be driver pathways. One approach to solve this problem is the propagation technique, in which the “influence” of each mutation disseminates through an interaction network, leading to the identification of a consistently perturbed group of genes (Fig 1A). HotNet, and its improved variant HotNet2, is a popular method in this class. It has been applied in the analyses of various cancer datasets to identify significantly mutated pathways in cancer [23,44]. HotNet accounts for local network topology and computes an influence network using a diffusion process from the source of heat (genetic alterations) within the network. Hofree et al. [45] adopted a network smoothing technique, which was previously used to infer disease association [32], for subtype stratification of patients in TCGA datasets. In their approach, the information on mutated genes for each sample was first propagated in the network (the network smoothing step). Then, non-negative matrix factorization, an unsupervised learning technique, was applied on the network with the smoothed mutation profiles to cluster cancer patients.

One of the challenges in designing and interpreting information propagation algorithms is related to the nonuniform distribution of node degrees in interaction networks. The existence of hubs in the network reduces distances between genes, implying that simple distance-based algorithms may not be sufficient to identify driver gene modules. Additional information, such as gene expression or genetic alterations [21], can be used to overcome the effects of shortcuts introduced by hubs and to limit the spreading of the influence signal into unrelated regions of the network [22,39].

One of the key insights contributed by network-centric approaches has been the extension of the concept of cancer drivers from individual genes to mutated pathways. Indeed, gene and gene products do not act in isolation, and the effect of a perturbation in a gene propagates through the interaction network, affecting the functions of other genes in its vicinity. By simulating the perturbation process in an interaction network, information propagation techniques are powerful tools that can recover true signals coming from different sources and even correct missing and uncertain information in incomplete interaction networks.

## Heterogeneity of Cancer and Personalized Networks

In complex diseases such as cancer, different cancer types can have different genetic causes, despite their phenotypic similarity in disease manifestation. This makes the development of methods that move towards finding personalized disease subnetworks a necessity. Such methods can lead to novel drug targets by capturing similarities and differences between patient subgroups.

Several approaches to capture the trade-off between individual differences and common principles build on the “set cover” problem, an optimization approach also known as the “hitting set” problem [46–50]. The basic formulation of the problem starts with assigning a normal or altered status to every gene for each patient. Such alterations might, depending on datasets, reflect a mutation, differential expression, differential methylation, etc. In a set cover formulation, a gene covers (or hits) the patients in which it has the altered status. Potential driver genes are selected to cover all patients while minimizing the number of selected genes. This allows different sets of altered genes to be chosen for different patients while preferentially choosing genes with higher alteration rates, helping to balance similarities and differences between patients. Note, however, that it is generally assumed that multiple mutated driver genes are required for cancer progression. Also, in the case in which alteration refers to differential gene expression, each cancer case will have many differentially expressed genes because of indirect impact. This leads to the multi-set cover problem, in which each patient is required to be covered not just by one but by multiple altered genes.

The original set cover definition does not account for relationships between covering elements (altered genes). Genes are connected via an interaction network, with interactions possibly weighted by their confidence or strength. Additional relationships between genes can also be implied by mutual exclusivity of mutations (which will be discussed in Taking Advantage of Cancer Heterogeneity: Utilizing Mutual Exclusivity of Genomic Alterations). To select gene modules that are representative of dysregulated pathways, network variations of set cover algorithms apply a set cover technique in the context of a gene interaction network so that the algorithms preferentially select genes connected in the network and find one or more gene modules covering patients. For example, DEGAS algorithm selects a connected subnetwork of genes covering patients multiple times so that the size of the selected subnetwork is minimized, aiming to identify a putative dysregulated pathway [51]. The algorithm finds one dysregulated pathway covering all patients (allowing a few outliers). However, this approach may not be optimal when there are different sets of pathways dysregulated in different patients. Acknowledging the possibility of multiple dysregulated pathways, the Module Cover algorithm (Fig 1B) finds multiple connected subnetworks (gene modules) that collectively cover all patients while minimizing the total cost of selected gene modules. The application of Module Cover to glioblastoma and ovarian cancer datasets revealed that different patient groups might be characterized by different combinations of dysregulated modules, which suggested that the selected modules could be used for subtype classification.

Module cover and heat diffusion-based algorithms share the common goal of finding dysregulated subnetworks. While the subnetworks obtained by both algorithms included frequently mutated driver genes, they differ in their treatment of rarely mutated putative drivers. Module Cover attempts to ensure that the selected pathways cover all patients so that driver genes covering only a small subset of patients are still captured. On the other hand, utilizing information flow can uncover some rarely mutated drivers, based on evidence that they might influence the same dysregulated genes. The methods also differ in which type of network is most suitable to use. Information propagation methods, when combined with physical interaction networks, can predict genes that mediate the flow of information. On the other hand, densely connected functional networks are natural in the context of Module Cover, which is designed to look for dysregulated gene modules that are functionally related.

Steiner trees are another classical graph-theoretical concept that is often utilized to find relationships between mutated genes. As with the Set Cover problem, several variations of the problem have been considered. The basic formulation of the Steiner tree problem is as follows: given a set of altered genes, interconnect them by a tree with the minimum cost using network edges and Steiner nodes (additional genes connecting the given altered genes). The prize-collecting Steiner tree (PCST) algorithm has been previously applied to find response networks [52–55], in which genes known to be associated with diseases are prizes and Steiner nodes are selected to collect the prizes with a minimum cost. Gitter et al. extended the PCST algorithm to obtain personalized Steiner trees for patients [56]. In personalized PCSTs, each sample can have a different PCST, but there is a penalty associated with differences between the personalized trees to maintain their similarity. This allows for finding trees that are optimal for individual patients but similar to each other. Gitter et al. reported that in an application to the basal-like TCGA breast cancer subtype, their method identified canonical pathways that significantly overlap with those uncovered by HotNet but also produce patient-specific pathways for different patients [56].

Both the Module Cover and personalized PCST algorithms find dysregulated subnetworks while considering cancer heterogeneity by approaching the problem from different angles. The former collects information from different patients simultaneously, aiming to discover gene modules while allowing individual patients to be covered by different gene modules. The personalized PCST algorithm, on the other hand, looks for a personalized network for each patient individually and uses information from other patients to bias the cost function and capture similarity between patients.

## Taking Advantage of Cancer Heterogeneity: Utilizing Mutual Exclusivity of Genomic Alterations

While cancer heterogeneity among patients makes uncovering genetic causes challenging, the principle that different genetic alterations lead to similar disease phenotypes might also be utilized to find previously unknown interactions between genes. It was often observed that mutations in cancer driver genes appear in mutually exclusive sets of cancer patients. One explanation for mutual exclusivity is that only one such mutation in a pathway is sufficient for cancer progression. A proposed alternative explanation is that mutually exclusive pairs may have a relationship of synthetic lethality or sickness, in which mutations in each gene separately promote cell growth but simultaneous mutation in both genes is lethal or detrimental. Note, however, that a synthetic lethal partner of a cancer driver does not necessarily have to be a cancer driver itself. Algorithms that predict synthetic lethality or sickness from mutational data typically infer mutual exclusivity patterns using additional information about gene expression patterns, effects on disease manifestation, and/or information from shRNA experiments in cancer cell lines [57,58].

Genes with mutually exclusive mutation patterns are often functionally related. This observation led the mutual exclusivity principle to be used as a tool to find cancer driver modules. Several computational methods have been developed to detect mutually exclusive gene sets [47,50,59–66]. For example, Ciriello et al. proposed MEMo (Mutual Exclusivity Modules), an approach that uses a permutation test to estimate the significance of mutual exclusivity and combines the results with known interactions, such as PPI, to find modules that are fully connected and show mutually exclusive mutational patterns [59,60]. Later they extended this idea by utilizing human signaling pathways to find groups of altered genes that are mutually exclusive and have a common downstream event [61].

Based on the assumption that mutual exclusivity can predict novel functional relationships, several approaches do not restrict the search space to gene pairs with known interactions but instead look for gene modules using only mutual exclusivity information of genomic aberrations [50,62–66]. Some methods were extended to detect multiple mutually exclusive groups of genes [67,68] or to refine mutual exclusivity models to account for temporal dynamics [69]. The algorithms in these types of approaches need to consider exponentially many combinations of genes, and rapidly increasing computation time is often a concern. Therefore, they typically focus on a limited number of candidate genes and modules of small sizes.

Given the utility of mutual exclusivity principle for uncovering important relationships between cancer drivers, one can also leverage datasets from multiple cancer types for integrated analysis. The TCGA Pan-Cancer initiative compiled datasets from multiple tumor types, aiming to identify the similarities and differences among them and to uncover cancer driving genes that are rarely mutated and may not be recognized in the small number of samples of individual cancer types [70]. For mutual exclusivity analysis in multiple cancer types, Kim et al. utilized TCGA Pan-Cancer datasets and introduced permutation test methods to differentiate mutual exclusivity within, across, and between cancer types [47]. They defined the “within” cancer type mutual exclusivity to occur when mutual exclusivity is observed for a given cancer type. Two genes are mutually exclusive “across” multiple types if combined signal is more significant than within type exclusivity in individual cancer types. Finally, the “between” type mutual exclusivity occurs when two genes are mutated in two disjoint sets of tissue types exclusively. Such scenario can occur when we deal with tissue type-specific drivers. Kim et al. found that while not all mutually exclusive gene pairs are functionally related, functionally interacting pairs are enriched with the across type mutual exclusivity relative to the between types mutually exclusivity [47]. These findings suggest that mutual exclusivity across multiple cancer types might facilitate the identification of cancer driver pathways dysregulated in multiple cancer types. Kim et al. incorporated the across type mutual exclusivity scores into the Module Cover algorithm (Fig 1B) and used the algorithm to identify common driver modules in Pan-Cancer datasets.

In another approach to analyze mutual exclusivity in the context of Pan-cancer data, Park et al. asked if some gene pairs are mutually exclusive in a specific cancer type more often. They found that some highly mutated gene pairs may have tissue type bias in mutual exclusivity patterns, which can be attributed to type specific interactions for the tissue type [71].

## Patient Similarity Networks

Modeling disease heterogeneity can also naturally start from the perspective of phenotypic similarity of individuals. Organisms or individuals whose phenotypes are determined by genomic elements can be represented as nodes and connected by edges if they have similar phenotypes. For example, Roque et al. [6] constructed a patient network based on the similarity of patients’ disease ontology, which was automatically extracted from their electronic records by using text mining approaches. Extracted phenotype information from psychiatric patient medical records was mapped to disease codes in the International Classification of Diseases ontology (ICD10), which contains codes for diseases, signs and symptoms, complaints, social circumstances, etc. Thus, each patient was represented as a vector using weighted significance of ICD10 occurrences. Afterwards, patient-patient similarity was defined by the cosine of the angle between every pair of patient vectors. Using a threshold of similarity scores, they obtained a network of approximately 1,500 patients that could be naturally clustered into subnetworks. They examined the features of these subnetworks and found that Schizophrenia and alcohol/drug usage were important features of several subnetworks.

Such a simple phenotype-based clustering approach provides valuable information but still has some shortcomings. For example, while the above-mentioned study found that several subnetworks have schizophrenia as one of their underlying features, it is not clear if the different subnetworks correspond to subtypes of schizophrenia or if phenotypic features unrelated to the disease define the subnetworks. In modeling heterogeneity of complex diseases, it is critical to find the causal relationships of disease phenotypes rather than simply clustering the phenotypes of patients. In the context of cancer, the cause is typically a tumor genotype, but other factors such as epigenetics, age, environment, etc., should also be considered when data permits.

**Fig 2:**
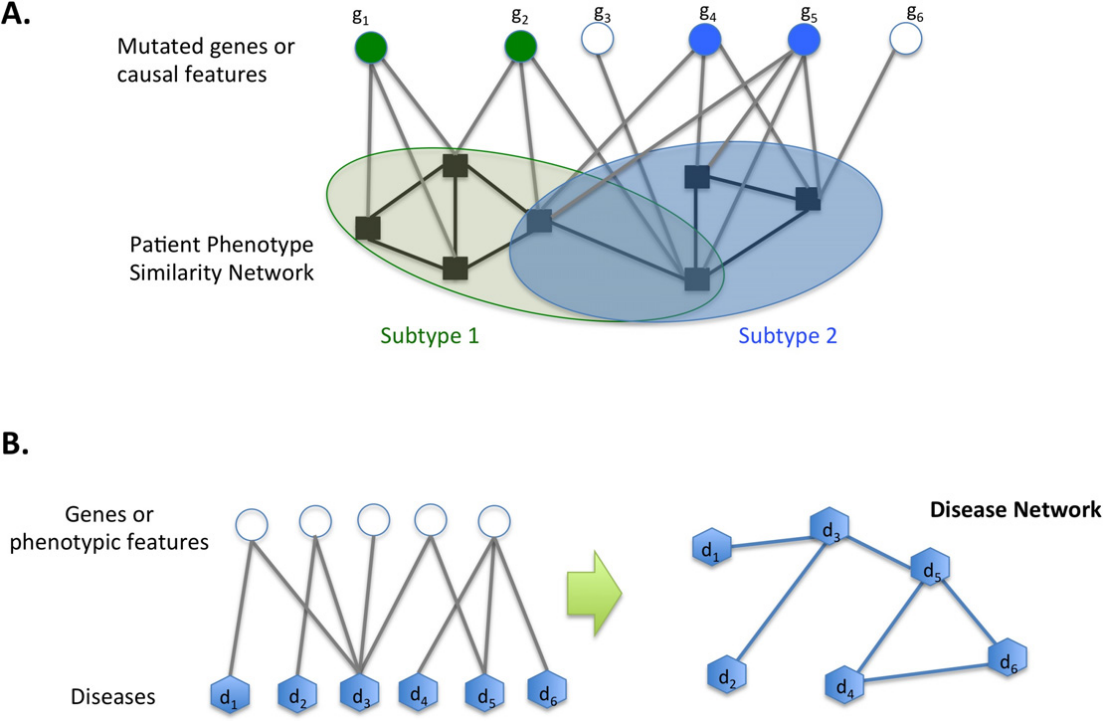
Other types of biological networks. A) Topic model utilizing a patient similarity network. The network guides to find disease subtypes and their features (in the figure, the mutations in genes g_1_ and g_2_ are selected features for Subtype 1, while Subtype 2 has mutations in g_4_ and g_5_). Patients can be represented as mixtures of multiple subtypes. B) Disease network. A disease network can be constructed based on shared disease genes or the similarity of disease phenotypes. For example, the disease network on the right has an edge between two diseases if they share the same disease genes or phenotype features.

The first approach to use a patient network to infer genotype–phenotype relationships was proposed by Cho and Przytycka [7]. By combining a patient similarity network and a topic modeling approach, originally designed to discover hidden semantic structures in large document collections (see [72] for a general introduction and [73–77] for biological applications), they developed and applied a probabilistic algorithm to analyze TCGA glioblastoma multiforme (GBM) data [78,79]. Specifically, the basic idea of a topic model is to identify topics so that each document is represented as a mixture of these topics. In a topic model for disease studies, patients are considered as documents, while putative causal features (mutations, copy number variations, etc.) of the patients represent the words describing the documents. In addition, gene expression was used to define phenotypic similarity between patients in the patient network. A topic model algorithm can then be applied to identify disease subtypes (corresponding to topics), and individual patients are modeled as a mixture of subtypes that best explain the patient similarity network. A patient similarity network is used to guide model construction so that phenotypically similar patients are assigned similar subtype mixtures (Fig 2A). In their analysis, the model inferred from the TCGA GBM data suggested that the previously proposed classification of GBM into four distinct subtypes [78] may be better explained with the classification into three basic subtypes (Proneural, Mesenchymal, and Classical) by representing the Neural subtype as a mixture of the Proneural and Mesenchymal subtypes [7]. The model also succeeded in identifying the underlying features that explain the subtypes. In summary, this approach makes it possible to uncover genomic aberrations that explain similarities and differences in patients’ phenotypes, identifying subtypes as well as dependencies between aberrations. Interestingly, the classification into three subtypes was also confirmed by applying iCLuster (an integrative clustering method based on a Gaussian latent variable model) [80]. It is worth noting that the technique used in this study is general and can be used to connect differently defined phenotype similarities, such as cancer stages or survival time, with a different set of putative causes, including changes in transcription factor binding site, methylation, DNA topology, and environmental factors. As opposed to two-step approaches that cluster patients and then find the underlying features, the topic model method constructs the subtypes models simultaneously with the subtype assignment and has the advantage of allowing each patient to be assigned a mixture of subtypes.

So far we have discussed using patient networks in which patient similarity scores are computed based on the combination of their phenotypic similarity features. An alternative approach was recently proposed by Wang et al., in which they constructed patient similarity networks independently for three different data types (mRNA expression, DNA methylation, and microRNA expression), then fused them into one similarity network [8]. More specifically, the Euclidean distances between every pair of patients were first calculated, and a scaled exponential similarity kernel was used to define patient–patient similarity matrices for each data type. In the network-fusion step, a nonlinear method based on message-passing theory that iteratively updated each network was used. Through this process, edges whose similarities are high in one or more networks are added to others, but edges whose similarities are low disappear. After some iterations, the process converged to a single similarity network. For five different cancers from TCGA, this similarity network fusion (SNF) method outperformed single data type analysis in identifying cancer subtypes and predicting survival time.

The two methods of utilizing patient similarity described above have their unique advantages. The topic model-based method can be thought of as a subtype-centric approach, as it allows probabilistic disease subtypes to be defined based on the frequencies of putative causal features while representing each patient as a mixture of these subtypes. On the other hand, the network fusion algorithm is a more patient-centric approach, which allows, for each patient, other individuals that are most similar to the patient in question to be identified and therefore enabling the information from neighboring patients to be transferred.

## Disease Similarity Networks

Disease similarity networks are a natural extension of patient similarity networks. Rather than considering individual patients, diseases are considered as nodes and edges are created based on similarities between the diseases. Considering diseases in the context of other related diseases can offer insights for better understanding their genotypic causes and further provide a way for repurposing existing drugs in similar diseases.

Similarity in disease networks may be measured based on various factors, such as shared disease genes [11,36,81], disease symptoms [82], and/or mRNA expression [9]. For example, Goh et al. [81] constructed a human disease network by first creating a bipartite graph based on disease–gene associations and then connecting diseases if they share the same disease genes (Fig 2B). Alternatively, disease phenotype similarity networks may be constructed based on phenotype similarities obtained from text mining [82].

Disease networks are often used in conjunction with a gene interaction network to improve the quality of disease-gene associations or to better predict disease similarities [12,13]. Menche et al. devised a metric to predict the similarity between diseases based on the degree of separation of disease gene modules in a gene interaction network [11]. They found that, in comparison to well-separated diseases, diseases with overlapping gene modules are significantly more similar in their expression patterns, symptoms, and have significant comorbidity. Suthram et al. obtained functional gene modules for diseases and used mRNA expression data to determine the status of the gene modules, creating a disease similarity map [9].

Overall, disease similarity networks are helpful for uncovering relationships between diseases, which in turn can shed light on finding common genetic causes among similar diseases. Another benefit of considering similarities and differences of diseases is that a large-scale disease interaction map can provide valuable guide for drug repositioning and help predict their side effects.

## Conclusions and a Look Ahead

Cancer heterogeneity poses a significant challenge for analyzing and interpreting cancer patient data. Cancer is now commonly understood as a disease of pathways rather than a result of defects in individual genes. Therefore, network-based approaches can be employed to find explanations for how different mutations result in similar disease phenotypes. Gene interaction networks have been used to identify defective pathways, classify subtypes based on subnetworks, and predict treatment and survival outcomes. In addition to gene networks, patient similarity networks are gaining importance and can offer different perspectives for understanding cancer.

Currently, networks used in cancer studies are typically static, and network topology remains fixed during the analysis while changes associated with diseases may be mapped as changes in properties of the nodes or edges of such network. However, interactions between biological entities may differ at multiple levels, depending on spatial and/or temporal conditions [83]. Recently, there have been some efforts towards modeling and analyzing dynamic interactions in the context of multiple time points [84,85] or tissue types [86].

Dynamic network analysis can help in deciphering fundamental mechanisms of diseases, such as increasing disease susceptibility with age. As a step in this direction, Faisal and Milenkovic exploited network dynamics to study age progression [85]. By combining a protein-protein interaction network with aging-related gene expression data, they obtained age-specific networks and analyzed global and local changes in network topology at different ages. They observed a number of proteins with changes in local network structures that are predictive of genes related to aging.

In cancer data analysis, utilization of network dynamics is still limited. Obtaining interactions in different tissue types and putting them together can advance therapeutics by predicting the effects of chemotherapy in specific tissues. For example, Greene et al. [86] used a support vector machine classifier, trained with tissue-specific networks, to re-evaluate significant genes from a genome-wide association study (GWAS). They showed that modeling complex diseases in humans using tissue-specific networks provided several insights into disease genetics and crosstalk, opening avenues for the discovery of molecular disease associations.

One of the most widely acknowledged problems with currently available gene networks is their noisiness. Physical interaction networks obtained from large-scale experiments are not only noisy but also incomplete. Relative to physical interaction networks, functional interaction networks are more complete but may lose specific information about directly interacting pairs because these networks integrate many different functional relationships together. Studies that focus more on mechanistic aspects of signal propagation may benefit from leveraging physical interaction networks, but they need to be aware of the limitations that these networks currently have. In contrast, analyses that focus on identification of broadly related groups of genes typically utilize functional networks, which cover a wide spectrum of interactions between molecules.

One of the main advantages of network-based approaches is their capability to uncover cancer-related associations, including genotype-phenotype relationships, despite the heterogeneity of the disease. Such large-scale associations can suggest potential treatment options and can generate testable hypothesizes, but at present, they can rarely provide full explanations of observed relationships. Indeed, current interaction networks not only lack context dependency but also cannot usually provide accurate mechanistic interpretations. It is anticipated, however, that as the coverage and information encoded in these networks improves, the predictive and explanatory power of network-based cancer analysis will increase accordingly.
